# Construction of a large scale integrated map of macrophage pathogen recognition and effector systems

**DOI:** 10.1186/1752-0509-4-63

**Published:** 2010-05-14

**Authors:** Sobia Raza, Neil McDerment, Paul A Lacaze, Kevin Robertson, Steven Watterson, Ying Chen, Michael Chisholm, George Eleftheriadis, Stephanie Monk, Maire O'Sullivan, Arran Turnbull, Douglas Roy, Athanasios Theocharidis, Peter Ghazal, Tom C Freeman

**Affiliations:** 1 Division of Pathway Medicine, University of Edinburgh, The Chancellor's Building, College of Medicine, 49 Little France Crescent, Edinburgh EH16 4SB, UK; 2 The Roslin Institute and Royal (Dick) School of Veterinary Studies, University of Edinburgh, Roslin, Midlothian EH25 9PS, UK; 3 Centre for Systems Biology, University of Edinburgh, Darwin Building, King's Building Campus, Mayfield Road, Edinburgh EH9 3JU, UK

## Abstract

**Background:**

In an effort to better understand the molecular networks that underpin macrophage activation we have been assembling a map of relevant pathways. Manual curation of the published literature was carried out in order to define the components of these pathways and the interactions between them. This information has been assembled into a large integrated directional network and represented graphically using the modified Edinburgh Pathway Notation (mEPN) scheme.

**Results:**

The diagram includes detailed views of the toll-like receptor (TLR) pathways, other pathogen recognition systems, NF-kappa-B, apoptosis, interferon signalling, MAP-kinase cascades, MHC antigen presentation and proteasome assembly, as well as selected views of the transcriptional networks they regulate. The integrated pathway includes a total of 496 unique proteins, the complexes formed between them and the processes in which they are involved. This produces a network of 2,170 nodes connected by 2,553 edges.

**Conclusions:**

The pathway diagram is a navigable visual aid for displaying a consensus view of the pathway information available for these systems. It is also a valuable resource for computational modelling and aid in the interpretation of functional genomics data. We envisage that this work will be of value to those interested in macrophage biology and also contribute to the ongoing Systems Biology community effort to develop a standard notation scheme for the graphical representation of biological pathways.

## Background

Macrophages and other antigen presenting cells (APCs) are present in high numbers in all tissues. They act as a first line of defence against pathogenic organisms playing a crucial role in co-coordinating the innate immune response to infection. Furthermore, it is being increasingly recognized that they not only play a central role in tissue homeostasis and development, but also in the aetiology and maintenance of pathological processes that underpin all infectious, inflammatory and malignant disease [1,2]. Whilst our ability to perform quantitative and qualitative measurements on the cellular components of the macrophage has increased massively, as has our knowledge on how they interact with each other, we have failed to convert these observations into detailed models of these systems. However, without such models we cannot hope to truly understand macrophages or indeed any other cell at a systems level.

Our primary interest has been to further our understanding of the macrophage signalling and effector pathways that orchestrate this cell's pivotal role in infectious and inflammatory disease. As with many systems, certain macrophage pathways are very well characterized whereas little is known about many others. Even where pathway domain knowledge does exist however, it is generally fragmentary and subjective. Therefore we set out to generate an integrated model of macrophage pathways of interest to us and in doing so we have faced one of the central challenges in pathway biology: How does one construct clear concise pathway diagrams of the known interactions between cellular components that can be understood by and useful to a biologist?

Decades of research on the functional activity of individual proteins and genes has revealed many insights into how these cellular components interact with each other to form the metabolic, signalling and effector effecter pathways that underpin life. Much of this work however remains locked inside the literature where specific insights into pathway function are subject to the semantic irregularities that come with their description by different authors. As a result, the details of a given pathway have traditionally been known only to a few experts in the field whose research is often focused on a single protein and its immediate interaction partners. Pathways are understood more generally by their description in reviews and diagrams produced on an *ad hoc *basis. If we are to escape this gene-centric view of biological systems, we must develop better ways to order and display our knowledge of protein interactions and the systems they form. Formalized diagrams act as a visual representation of the interactions between cellular components and provide a valuable resource for modelling network structure and the dependencies between components [3]. In addition, pathway models are an invaluable resource for interpreting the results of genomics studies [4-10], for performing computational modelling of biological processes [11-15] and fundamentally important in defining the limits of our existing knowledge. Large integrated diagrams of metabolic pathways have been available for many years, for example Gerhard Michal's classic biochemical pathways wall chart first published by Boehringer-Mannheim in 1968. Such pathway diagrams are inevitably complex, but potentially liberate the user to explore the interconnectivity between what might be seen as separate pathways and get an overview of topology of the system as a whole. In contrast, the assembly of detailed diagrams of signalling pathways as integrated networks rather than a series of disconnected views has been little explored.

In recognition of the importance of pathways, many efforts have been made to collate pathway knowledge, together with information derived from large-scale interaction studies and literature mining, into public and commercial databases [16-25]. These offer searchable access to pathway diagrams and interaction data derived from a combination of manual and automated (text mining) extraction of primary literature, reviews and large-scale molecular interaction studies. Whilst invaluable and in many ways the best we have, a major problem with these efforts is that the information content of these diagrams is frequently limited and visualizations of these systems are of variable and often poor quality; Pathway components are often labelled using inconsistent nomenclature systems and depicted using a variety of shapes (glyphs) to illustrate component 'type'. Furthermore, notation schemes used for pathway diagrams to depict one molecule's interactions with another are not standard and are often limited in their ability to convey the exact nature of the relationship between components. Finally, pathway diagrams are generally highly subjective reflecting the curator's bias, such that two diagrams depicting the 'same' pathway may share little in common. Together these factors commonly result in uncertainty as to what exactly is being shown. The diagrams thereby fail to fulfil their basic purpose - to provide a comprehensive and unambiguous picture of what is known about a pathway. In an effort to address some of these issues, a number of groups have suggested notation schemes for drawing 'wiring diagrams' of cellular pathways [26-29].

Over the past four years we have been constructing process diagrams [26] of pathways important in regulating macrophage immune biology and known to be activated in these cells during infectious and inflammatory disease. In constructing these graphical models which encompass a diverse range of biological pathways and concepts, we found it necessary to refine the Edinburgh Pathway Notation (EPN) scheme as previously proposed [28,30]. The current 'modified' EPN scheme has been arrived at through extensive use and testing. A specification document describing the complete list of mEPN symbols and rules for their correct use can be found on http://www.mepn-pathway.org[31]. The macrophage pathway model presented here, drawn using the mEPN scheme, explores some of the challenges associated with meeting the various demands of a pathway diagram. We hope it will prove to be a useful resource for macrophage biologists, as well as an important contribution to the debate on pathway notation and depiction.

## Results

**Figure 1:**
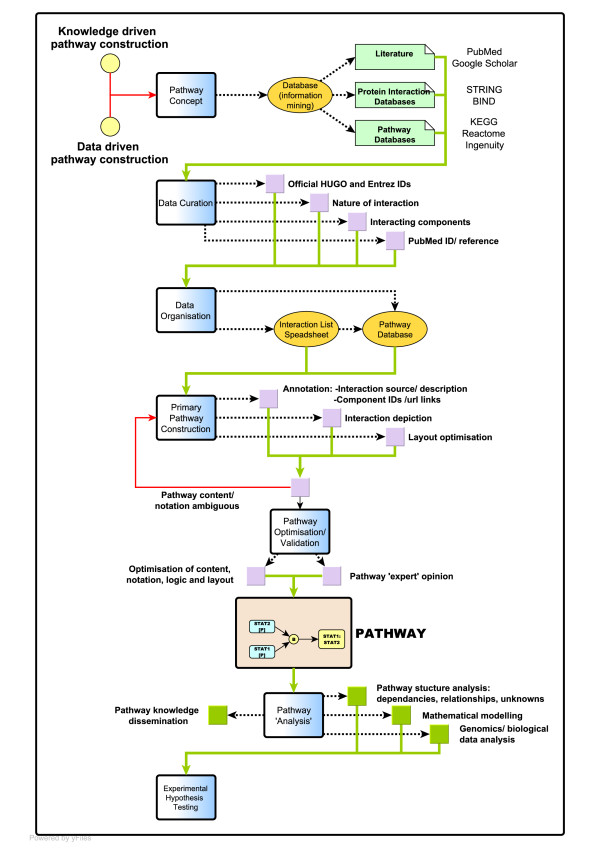
**Pathway construction workflow**. A workflow diagram summarizing the main stages of pathway assembly from concept to final diagram. Blue boxes portray the pathway construction phase. Each phase embodies a number of tasks (shown as lilac boxes or yellow-ellipses for data storage and processing), which when completed lead to progression towards the next stage of pathway construction (connected by green arrows). Red arrows indicate feedback to a previous construction phase. Lilac boxes describe the construction steps required pre-pathway assembly, whereas green boxes are linked to post-construction phases and describe the possible applications of the constructed and validated pathway diagram.

The work described here follows in the footsteps of earlier efforts to construct process notation diagrams of macrophage pathways [30,32]. In the course of the current effort we have sort solutions to issues associated with the depiction of a variety of different biological systems, combining diagrams from multiple curators and the layout and integration of a large network model of these systems. A schematic view of the steps involved in the assembly of this pathway is shown in Figure 1.

### Integrated Pathway Diagram

**Figure 2:**
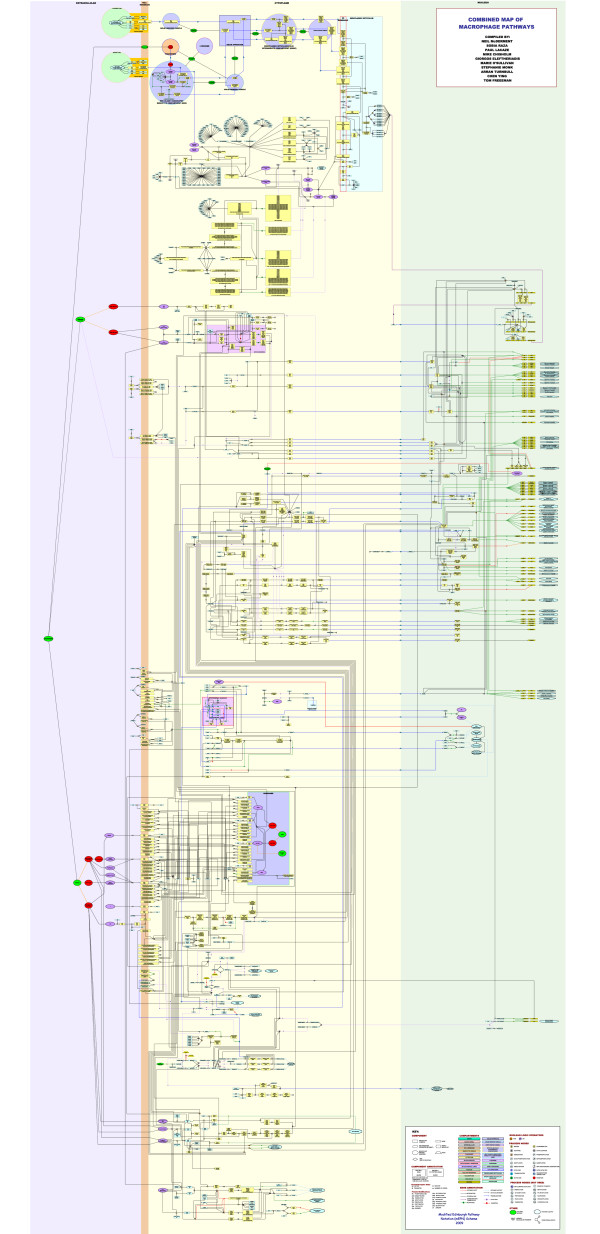
**Integrated pathway diagram of innate immune and macrophage activation pathways**. The modified Edinburgh Pathway Notation (mEPN) scheme is used to describe the interactions of signalling pathways active in the macrophage. A total of 2,172 components in this network are connected by 2,553 edges. Components include 496 unique proteins, the complexes formed between them (412), 181 genes/DNA/promotor regions, in addition to other molecular species (e.g. pathogens, drugs, RNA) and the nodes representing the processes in which the components are involved. Components are arranged to reflect the location in which they are active and background colour is used to distinguish between different sub-cellular locations.

**Figure 3:**
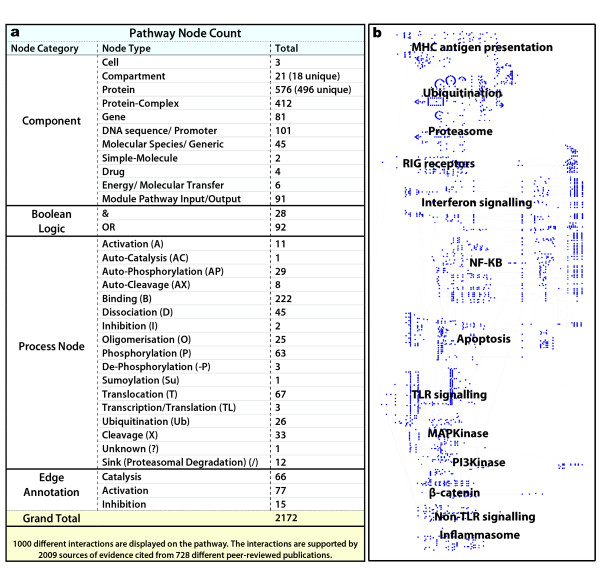
**(a) Breakdown of node class in the integrated pathway diagram and (b) Key to the pathway layout and content of the integrated diagram**. **(a) **Components, Boolean Logic, Process Nodes and Edge Annotation form the category of possible nodes. A detailed breakdown of the number of each type of node in each category is given. **(b) **The key reflects the approximate location of the different pathway modules depicted in the integrated diagram (Figure 2). Ideally pathways with high connectivity and sharing identical components are spatially located in close proximity.

The pathway diagram presented here (Figure 2 and additional file 1) is a consensus view of a number of pathway modules assembled based on our interpretation of the literature describing these systems. A given interaction between components of the pathway may be supported by evidence derived from one or more publications and a publication may provide evidence supporting more than one interaction. A total of 1,000 different interactions have been recorded, supported by 728 different original papers and reviews (see additional file 2 for a table with the full list of interactions and supporting publications). The network diagram is comprised of 2,172 nodes connected by 2,553 edges. The diameter of this network (maximum distance from one node to another) is 58 and there is an average node connectivity (number of inputs/outputs) of 2.37 (max 37). A detailed breakdown of the class (type) of the nodes that make up the diagram is shown in Figure 3a. Briefly, 496 unique proteins are represented in the diagram many of which are shown to go on to be modified into different forms or bind together resulting in 412 different complexes. 81 genes are shown to be transcriptionally regulated by these pathways based on known associations between transcription factors and target genes. The interactions between these components are represented by 552 process nodes, 120 Boolean logic operators and 158 edge annotations. The pathway is drawn using the mEPN scheme, a full description of which can be found on http://www.mepn-pathway.org and in Freeman *et al.*, 2010 [31] and this and macrophage-related pathways are available through http://www.macrophages.com.

### Description of the Biological Content of the Pathway Diagram

The pathway diagram (Figure 2 and additional file 1) incorporates detailed views of some of the best characterized pathways associated with macrophage-specific biology, as well as some that are generic, but in some way linked to the activity of these cells. Figure 3b shows the approximate location of the different pathway modules within the diagram. The size of the diagram requires it to be ideally viewed on a computer. Every effort was made to arrange modules so those with shared nodes and high connectivity are located in close proximity, however given the issues in depicting information on this scale, 'ideal' arrangement of components is challenging. The interactions between components and limited views of the pathways they form have been described in detail in the literature used to construct this diagram (see interaction table, additional file 2). An overview of the pathway biology depicted by the diagram follows.

### Macrophage Pathogen Receptor Systems

**Figure 4:**
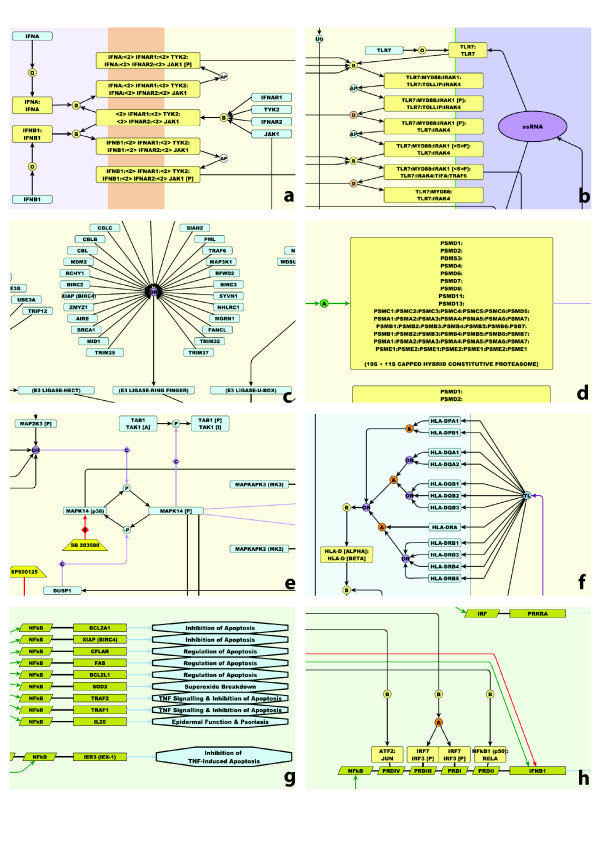
**Snapshots from the integrated macrophage pathway diagram. (a) Activation of the interferon type-1 receptor through its interaction with interferon-α (IFNA) or interferon-β (IFNB1)**. In each case binding of the ligand causes autophosphorylation of JAK1 which eventually leads to the type1-interferon response (not shown). **(b) Activation of TLR7 by single stranded RNA in the endosome**. This sequential multistep process involves binding events, autophosphorylations and dissociations steps. **(c) E3 ligase system**. Up to 500 proteins may potentially function as E3 ligases and here the well documented members are shown. **(d) Depiction of the proteasome**. In some cases it is useful to lay out the subunits of a complex to reflect the complexes known structure. Represented here are the layers of the proteasome's barrel structure and cap. **(e) Activation of MAPK14 (p38)**. Phosphorylation of p38 is reversible; numerous kinases will phosphorylate p38. p38 is dephosphorylated by DUSP1 and inhibited by the specific inhibitor SB203580. **(f) Combinatorial assembly of the MHC class 2 HLA-D (alpha/beta) complexes**. The & and OR Boolean operators indicate the combinatorial assembly of HLA-D (alpha/beta) complex from different classes of MHC class 2 proteins. **(g) Genes activated by NFKB1 (p50):RELA (p65) complex**. A number of genes activated by the binding of the p50:p65 complex to known NFKB elements in their promoter. In each case the likely functional consequence of this activation is shown as a pathway output. **(h) Regulation of *IFNB1 *expression**. Shown are the known promoter elements and factors that bind to them leading to *IFNB1 *expression.

Macrophages are equipped with a complex array of pattern recognition receptors (PRRs) that bind a varied assortment of pathogen-associated ligands. Perhaps the best studied of these are the membrane-associated toll-like receptors present on the cell's surface (TLRs 1/2/4/5/6/10) and lining their endosomal compartments (TLRs 3/7/8/9) [34,35]. The receptors commonly form complexes comprised of 6-8 protein subunits which undergo a series of phosphorylation, dissociation and binding events following engagement of the receptor with their respective ligand class. As with all such receptor-ligand interactions shown in the diagram, each successive stage in the formation and activation of the receptor complex is explicitly shown (Figure 4a &4b). TLR's are comprised of two functionally significant domains; one for recognizing specific pathogen associated molecular patterns (PAMPs) and one for recruiting signalling adaptor proteins following binding of an appropriate pathogen-derived ligand. The pathogen recognition domains of different TLR's are structurally highly variable [36], thereby allowing the recognition of diverse pathogen-derived molecular species ranging from viral double- and single-stranded RNA [37,38] bacterial flagellin [39,40], lipopeptides [41,42], lipopolysaccharides [43,44], and bacterial and viral CpG motifs [45,46]. In contrast, the internal domains tend to be more conserved, reflecting the ability of different TLR's to recruit the same adaptor proteins; in particular MYD88, IRAK4, IRAK1, TOLLIP, TIFA, and TRAF6 are common to most of the TLR receptor complexes. The use of common adaptor proteins by many of the TLR complexes represented a significant challenge in depiction, with many edges emanating out of each adaptor molecule. Much effort was therefore put into layout of this system so as to provide visual clarity. A comprehensive and systematic effort to depict TLR signalling has been reported elsewhere [31] however this was not used in the construction of the current view of TLR signalling. These receptors ultimately activate a number of downstream signalling pathways including the NF-κB, IRF (interferon regulatory factor) [47] and MAPKinase, ERK, and JNK signalling [48].

Also represented here are 9 non-TLR cytoplasmic PRRs, including the NOD (nucleotide-binding oligomerization domain)-Like Receptors and RNA helicases, whose role it is to detect endogenous stress signals and intracellular pathogens. NOD-like receptors (NLRs) can be broadly divided into various classes, the NODS, NALPS and other types of NLRs, based on their protein domain structures. Bacterial associated PAMPs (e.g. flagellin and various classes of peptidoglycans) are recognised by NOD1, NOD2 and NLRC4 (IPAF1) which activate the NF-κB and MAPKinase pathways. NOD1 can also lead to the cleavage of IL1B into its active form. The NALPS (NLRP1, NLRP2, and NLRP3) detect a range of stress signals such as K^+ ^efflux, DNA, ATP or membrane damage, often collectively referred to as danger-associated molecular patterns (DAMPs). Once activated by DAMPs the receptors form oligomers with inflammatory caspases (CASP1/5) and in doing so activate the cleavage of the caspases [49]. The active complexes are known as 'inflammasomes' owing to their ability to cleave and activate interleukin proteins, and are crucial mediators of the inflammatory response. Although three NALP receptors have been depicted up to 14 different NALPS have been reported [50,51]. Finally, RNA helicases are responsible for the intracellular recognition of viral single stranded and double stranded RNA. The diagram shows DDX58 (RIG-1) and IFIH1 (MDA5) which recruit factors via their CARD domains and eventually initiate anti-viral gene expression by activation of the NF-κB system. The ZBP1 protein was recently characterized as a sensor of viral DNA [52] and activates the IRF3 transcriptional pathway. As such the PRR systems depicted represent a comprehensive view of these receptors and the signalling pathways they activate. However the diagram still lacks other known macrophage PRRs, including the surface mannose receptor, secreted receptors e.g. those belonging to the complement system and the recently described DNA receptor AIM2 [53-56].

### Cytokine Activation Pathways

The diagram also describes a number of the main cytokine signalling systems active in macrophages. These include the interferon (type 1 - IFNA/IFNB and type 2 IFNG), interleukin 1B (IL1B), TNF, TNFSF10 (TRAIL), TNFSF13B, FASLG and CD40LG signalling pathways. In each case these have been depicted starting from their interaction with their receptor complexes (Figure 4a) through to the activation of their downstream signalling and effector pathways. Interestingly, the expression of a number of these ligands is activated by PRR pathways (e.g. IL1B, IFNB) producing autocrine feed-forward loops. *IFNB *is perhaps one of the best studied genes in the whole genome in terms of its transcriptional regulation [35,57-59]. It is also one of the primary targets for a number of the macrophage PRR activation pathways described above and we therefore constructed a detailed model of its regulation (Figure 4h). Part of the reason behind this was also to grapple with the issues with depicting transcriptional networks and we believe the solution arrived at should work for other systems. In the current diagram however, we have generally chosen not to depict the links between cytokine gene activation (or indeed between other genes) and their respective proteins (via translation). The depiction of these edges adds to the visual complexity to the diagram. For modelling purposes however these connections can be added.

### Apoptosis (Programmed cell death)

A potential output of the innate immune response is to culminate in host cell suicide (apoptosis) thereby potentially limiting further reproduction of pathogenic organisms such as viruses. Two major routes of apoptosis execution have been identified, termed the intrinsic and extrinsic pathways. The intrinsic pathway is activated as a result of stress signals detected within the cell, for example, penetration of a viral pathogen into the cell or UV light induced DNA damage. Extrinsic apoptosis on the other hand is triggered by extracellular death-signalling ligands (FAS, TNFSF10 (TRAIL), TNF which are also members of the cytokine activation pathways) binding to the cell membrane receptors. Both intrinsic and extrinsic pathways activate a number of the caspase family of cysteine proteases. The initial caspases to be activated are categorized as initiators, (CASP2/4/6/8/9/10) and are capable of cleaving downstream executioner caspases, specifically CASP3 and CASP7. Caspases 3 and 7 initiate the series of events that directly lead to the morphological changes in a cell associated with apoptosis by the cleavage or inactivation of an array of molecules including, structural proteins, DNA repair proteins, and anti-apoptotic proteins.

### Signal Transduction and Transcription Factor Networks

The NF-κB (nuclear factor kappa-light-chain-enhancer of activated B cells) family of transcription factors are pivotal in the regulation of a wide variety of biological processes [60-63]. This includes many aspects of the innate and adaptive immune response, as well as the regulation of a diverse range of stress-related stimuli [64]. 5 different NF-κB proteins (REL (c-Rel), RELB (REL), RELA (p65), NFKB2 (p100 or p52), NFKB1 (p105 or p50) have been identified and these form a variety of homo- or hetero-dimers resulting in an array of different NF-κB complexes. Previously [30] we described the activation of two of the best-characterized NF-κB dimers; NFKB1:RELA (also known as p50-p65) and NFKB2 (p52):RELB, often referred to as the canonical and non-canonical pathways, respectively. However, we soon realized that this diagram was a rather naïve view of the NF-κB system and we set out to explore the literature on this system in greater detail. These efforts have resulted in our depiction of 14 different NF-κB dimers formed from combinations of the five NF-κB proteins. In addition to the 14 dimers, some NF-κB complexes form further complexes with a number of accessory proteins (NFKBIA/B/E/Z, BCL3, HMGA1, CREBBP, HDAC3, NCOR2) and together with their regulation by multiple phosphorylation events, give great diversity in the form and control of this important class of transcription factors. This goes someway to explaining the pleiotropic effects regulated by this system [65]. It is unlikely however that all the possible NF-κB systems depicted are active in the macrophage or indeed any other single cell type. NF-κB signalling is often cited in loose terms in literature with little reference to the exact NF-κB complex active in any given situation. The pathway diagram presented here demonstrates the complexity of this system and underscores the need for acknowledging the range of possibilities beyond the canonical and non-canonical NF-κB pathways.

As mentioned above, phosphorylation is a key element in the activation process of many of the NF-κB complexes. In the pathway, dimers of the core proteins may bind to NFKBIA, NFKBIB or NFKBIE, a group of I-kappa-B or NF-κB inhibitor proteins. When bound to their inhibitor, the NF-κB complexes are restricted to the cytoplasm. Upon stimulation the I-kappa-B proteins are phosphorylated, leading to their ubiquitination and eventual degradation, and the release of the active NF-κB complex. Dissociation of the inhibitors exposes the nuclear localization domain on the NF-κB complex causing it to translocate to the nucleus where it can modulate transcriptional activity of target genes [66,67]. Other NF-κB complexes (which are not bound to inhibitors) are activated following cleavage into smaller DNA-binding subunits. This is induced by stimuli phosphorylating the complex leading to its ubiquitination and subsequent processing of one or more of their subunits into smaller DNA binding peptides e.g. NFKB2 is processed from p100 to p52. Currently there are 34 genes shown on the diagram as the transcriptional targets of NF-κB signalling (Figure 4g). In reality this is only a small percentage of the known NF-κB targets [68]. Furthermore, the representation of the transcriptional regulation of these genes is almost certainly a gross over simplification, as there are likely to be other transcription factors acting in concert with NF-κB to modulate gene expression.

The pathway diagram presented here also includes preliminary views of some of the kinase signalling pathways known to be associated with macrophage activation. These include the MAP2K1/2 (MEK1/2)-MAPK3/1 (ERK1/2) cascade known to be activated by TLR signalling and the MAPK8 (JNK)-JUN (AP1) and MAPK14 (p38) (Figure 4e) cascades activated by NOD1/2 and TLR signalling. These pathways are clearly important to macrophage activation and are known to influence a range of different processes such as cell differentiation, cell cycle, phagocytosis and apoptosis. However, due to their apparent parallel and overlapping functions and their involvement with so many cellular processes, a precise understanding of their relative contributions to macrophage biology is unclear.

### Antigen Presentation and Related Pathways

Antigen presentation is not exclusive to macrophage biology but equally central to it. We have attempted to depict the MHC class I and II pathways from either the degradation of cellular proteins or the phagocytosis of pathogens, to the presentation of cellular/pathogen peptide antigens to CD8 cytotoxic T-cells or CD4 T-helper cells, respectively. In order to achieve this however, we found it necessary to construct diagrams of the ubiquitination pathway (due to it role in tagging cellular and pathogen proteins with ubiquitin) and proteasome formation (due to the role of the proteasome in the digestion and/or processing of ubiquitinated proteins). These latter two systems are also crucial to many aspects of cell biology, being responsible for the activation and/or degradation of many cellular and pathogen proteins alike. Depiction of these systems proved to be challenging. In the first instance, whilst our view of phagocytosis is greatly simplified, we had to find ways to show the passage of key molecules in the antigen presentation pathways from their assembly in the endoplasmic reticulum (Figure 4f) to their transport to the phagosome via the golgi and intermediate endosomal compartments. This required us to show events both at the molecular level, as well as the transition of compartments in which they reside from one state to another. In this case nodes representing pathway modules have been used to link one compartment to another indicating a series of vesicular transitions and fusions; complex processes in their own right. Another challenge was the depiction of the ubiquitination pathway. In short, proteins are tagged for degradation or cleavage through their binding to E3 ligases. Each E3 ligase or E3 ligase complex binds specific protein targets. We have shown a number of classes of these molecules i.e. the HERC/HECT, ring finger, U-box and SCF E3 ligases, encompassing over 80 proteins in total (Figure 4c). However many more proteins are thought to be associated with this role, perhaps as many as 500 [69,70]. To add to this complexity there are 36 known E2 ligases and 6 E1 ligases which add further specificity to this system. Clearly it would be impossible to show each individual protein and their associated E3 ligase passing through the ubiquitin pathway (even if the details were known) and therefore it has been depicted as a generic process; proteins bind E3 ligases, which provide a scaffold for ubiquitin transfer from E2 ligases, resulting in the ubiquitinated-protein being presented subsequently to the proteasome for processing. When a protein in the pathway is ubiquitinated we show this using a process node with the symbol Ub, which is essentially a short cut to showing this process. In our original pathway [30] we depicted the proteasome as a single node responsible for the cleavage and activation of ubiquitinated NF-κB complexes. As we looked further into the nature of the proteasome however, we began to appreciate that there is not just a single proteasome but at least 5 specific proteasome complexes; the 26S, 11S capped and 19S+11S capped hybrid constitutive proteasomes, and 11S and 19S+11S capped hybrid immunoproteasomes [71,72]. In an effort to show something of the structure of these large barrel-like complexes, we chose to depict the layered arrangement of core subunits into four stacked rings (7 subunits per ring/layer), where appropriate capped with other subunits that form the regulatory particles (Figure 4d). By linking the proteasomes with the generic model of the ubiquitination pathway we have also attempted to show each proteasome's preference for the cleavage of a specific class of peptides. However in doing this, it is not possible to show whether the given output of a proteins ubiquitination and cleavage is an activated peptide, a peptide for antigen presentation or the complete destruction of the protein. Where a protein in a pathway is degraded we have used the sink glyph (Ø) as a short cut to indicate that the protein has been removed from the system by proteasomal degradation.

### Compatibility of mEPN Pathways with Other Pathway Analysis Tools

The pathway model presented here is primarily designed to function as a computation resource. Its size as well as the fact that additional information is available through mouse-over or hyper-linked from it, means that it is best viewed on a computer. The diagram has been constructed using the freely available program yEd graph editor (yFiles software, Tubingen, Germany), a general purpose tool designed for the depiction of network-based diagrams. The standard file format used with this program is .graphml which is also supported by other network/pathway editors [73-76]. In this format pathways are available for editing and expansion or alternatively using the yEd editor, can be exported from the program in a number of image (.jpeg, .png, .pdf) and exchange formats (.tgf, .gml, .ygf, .xml, .html). The pathway is also provided as .pdf and .html formats (additional files 3 &4). In order to enhance options for the display, analysis and integration of these pathways with other data types we have recently implemented the import of .graphml files into Biolayout *Express*^3D^, a network analysis tool co-developed by our group [76,77]. This program supports a range of other network analysis features and is suited for working with small or large networks derived from other data sources. A 'layout' file has been provided (additional file 5) such that the diagram can be viewed in this tool as either a conventional 2D or 3D network diagram, in both cases using the node co-ordinates from the .graphml file (although polylines are not supported). Alternatively the diagram can be viewed in 3D using a modified Fructerman-Rheingold organic layout algorithm [78]. A notation system consisting of 3-D shapes is applied in the 3-D view of the pathway [31] (also see http://www.mepn-pathway.org). With the ever increasing amounts of interaction data it becomes more evident that an extra dimension will be valuable for the visualisation of large pathways and eventually an in silico cell. The BioLayout *Express*^3D ^interface also supports the follow through of connectivity in pathways such that the parent or children nodes of a given selection can be highlighted and selected nodes hidden or isolated.

**Figure 5:**
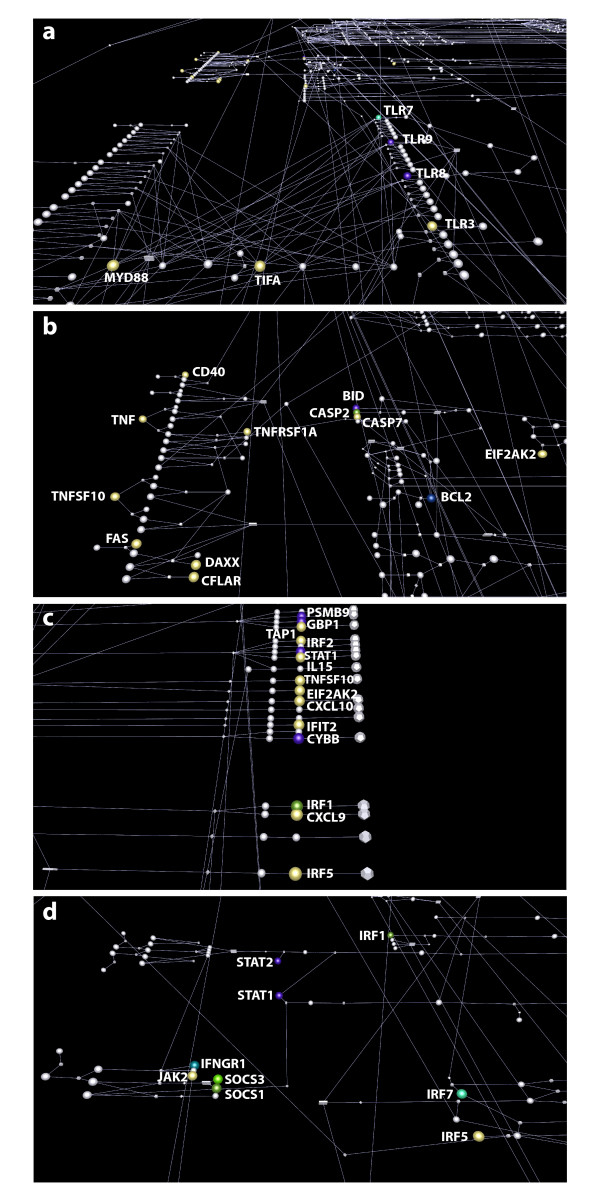
**Overlay of Ifn-β timecourse analysis onto the macrophage pathway using BioLayout *Express***^**3D**^. Views of regions of the macrophage pathway where components were found to be regulated Ifn-β. Components regulated at the level of gene expression are enlarged and coloured according to their cluster membership. All non-regulated components and other nodes are grey. **(a) Regulation of Toll Like Receptor Signalling**. All endosomal TLRs, Myd88 and Tifa genes are induced from 1 h, their expression being maximally up-regulated at 4-8 h post-Ifn-β treatment. **(b) Regulation of death-receptor and apoptosis signalling**. The expression of all regulated components in this module rises from 1 h and reaches a maximum 2-4 h post-treatment. In contrast, the expression of the anti-apoptotic gene *Bcl2 *is suppressed at 4 h. **(c) Regulation of the transcriptional targets of interferon signalling**. 7 genes (shown in yellow) belong to cluster 2 and their expression induced from 1 h, becoming maximal at 4 h. 4 genes (in purple) were in cluster 3, with expression rising from 1 h and remaining maximal throughout 4-8 h post-treatment. *Irf1 *(in green) reaches maximal expression at 2 h. **(d) Regulation of type I and type II interferon signalling**. All receptor components, except *Ifngr1*, belong to clusters of induced genes. *Socs1 *and *Socs3 *are induced and reach maximal expression at early time points (1-2 or 2 h).

Pathway diagrams are frequently used as an aid to the interpretation of experimental data e.g. gene expression analyses, proteomics screens whereby the results of these studies are be overlaid on top of pathways to provide context to the findings. To facilitate these analyses we have recently implemented an "import class-sets" functionality into Biolayout *Express*^3D^, allowing lists of genes of interest and/or annotations to be easily exported directly from the tool and identified on the pathway. Figure 5 and additional file 6 demonstrate the use of the "import class-set" function to highlight pathway components regulated in response to a timecourse of Ifn-β treatment of mouse bone marrow derived macrophages (GEO accession GSE20403). Lists of co-ordinately expressed transcripts from the mouse expression analysis were saved as a class-set file and imported onto the 'human' macrophage pathway diagram using the tool. This approach allows the rapid visualization and identification of those pathway components that are regulated by Ifn-β. 95 pathway components matched Ifn-β regulated transcripts, 77 were present in clusters representing up-regulated genes and 18 were present in down-regulated gene clusters. Additional file 6 provides the BioLayout *Express*^3D ^file of the macrophage pathway with the regulated genes enlarged and coloured according to their cluster membership. The pathway was inspected for regions of regulated components and some of the interesting observations are shown in Figure 5.

In the ways described above, the diagram presented here represents a detailed consensus view of a range of pathway systems that are of interest to and the subject of ongoing research into macrophage biology. It has been designed to be easily accessible, distributable and can be modified by end users to suit their interests or knowledge-base. Finally, we continue to develop software to facilitate the use of such resources for pathway modelling and interpretation of genomics data.

## Discussion

Interest in 'pathway biology' has never been greater as we struggle to comprehend cellular systems from a combination of targeted studies and the deluge of data flowing from 'omics platforms. This is reflected by the escalating efforts to assemble pathway diagrams [30,32,33,79,80], develop standards for depicting pathways [26-28,81], software to support their construction [82-84] and exchange [85-87], and the development of approaches to model and predict pathway behaviour [13,88,89]. Whilst arguably there is no such thing as a pathway only one big integrated network of molecular interactions, it is still useful to think in terms of pathways as being connected modules of this network. As such a pathway may be considered to consist of a specific biological input or event that initiates a series of directional interactions between the components of a system leading to an appropriate shift in cellular activity. As we begin to appreciate the potential complexity of these molecular networks, there is increasing interest in modelling pathways in order to expand our understanding of biology from the traditional gene-centric view of life, to a systems level appreciation of biological function.

The pathways described here are of central importance to understanding macrophage biology and therefore innate immunity, and the diagram provides a consensus view of these systems. This is not to say that the model is either viewed as complete or necessarily even correct, but only as a working model. As such it has been designed with the idea that it will need to be modified and expanded based on new publications, experimental observations or deeper insight into specific systems. All of the pathways depicted are reasonably well characterized and as such there is a relative abundance of information on them from a wide variety of sources. What we lacked prior to this work was a way of collating our understanding of these pathways and integrating this view with the abundance of data generated on these cells by ourselves and others. Our aim has therefore been the creation of a pathway diagram that graphically reflects the current view of a pathway system in a visibly intuitive manner. In so doing we wished to create a resource for data integration, pathway modelling and hypothesis generation. In order to achieve these objectives we found it necessary to modify both the PDN [26] and EPN schemes [28] for pathway depiction. Our original diagram [30] acted as our framework for the current effort helping to highlight the many gaps in our understanding and together with developing interests in macrophage biology, helped to prioritize areas for future modelling. Modelling of the pathways continued to be based on labour-intensive curation of the literature. Post-graduate students were given an area of biology to examine, and all the resources for researching the literature and depicting their chosen pathway module. Regular debates on the progress of the pathway models were held, and through this process deficiencies were plugged in the graphical depiction of events, pathway content, notation, component labelling and the recording of the supporting information; a process which in itself was highly informative. An important point is that the diagrams can be shared and understood by all those familiar with notation, and as a result all the work presented here has been subjected to form of internal critiquing. However, each new area of biology included in the current diagram has presented its own problems in layout and concept representation. As a result there has been subtle but almost constant re-evaluation of various aspects of the notation scheme and as we have dealt with new issues in the depiction of different systems. We are now satisfied that mEPN scheme has matured to the point where we foresee little need to change the majority of the notation scheme presented here (see Freeman *et al.*, 2010 [31] and http://www.mepn-pathway.org), although clearly the modelling of other systems and ideas from others may present a case for further modifications.

Pathway diagrams are a well established tool in our effort to interpret and explain results from functional genomics investigations. Overlay of results, usually from studies of the difference between one biological state and another, on top of pathway diagrams allows the investigator to visualize and link observations to defined pathways. BioLayout *Express*^3D^, a network analysis tool developed by us, provides a powerful approach to visualize and analyze 'omics data from a variety of sources [77]. We have recently implemented the import of .graphml files into BioLayout *Express*^3D ^and the tool now supports the visualization of pathway diagrams as 3D or 2D networks [76]. A parser automatically converts the mEPN notation into the equivalent 3D notation scheme and can use the diagrams original node co-ordinates to layout the pathway. We have now also implemented the ability to export analyses from one dataset e.g. clustering of microarray gene expression data and import and overlay these analyses on to another network. As we have used standard gene nomenclature in the assembly of this pathway it is possible to map directly between gene identifiers from data to genes/proteins in the pathway. Analysis of the transcriptional response of mouse bone marrow derived macrophages (BMDM) to Ifn-β stimulation has been used here as an example (Figure 5 and additional file 5). In practice any number of lists with annotations can be imported as class-sets onto the pathway and one can envisage how this would facilitate the comparison of numerous data sets in the context of the macrophage pathway. Although the concept of data mapping onto pathways is not new and is supported by other pathway resources [6,19,90] these pathways suffer from a number of issues pertaining to the lack standard graphical notation used to depict them. Furthermore the nature of the pathway presented here (in terms of scale, detail, formalised notation, range of pathways covered and integrated nature of their presentation) presents a valuable additional resource for those interested in macrophage biology or any of the pathways covered. Clearly the better and more extensive the pathway diagrams are the easier it will be to provide a working hypothesis on the interpretation of data. Increasingly, it is now experimental data that is helping to refine existing pathway models and observations that we do not understand that are driving our current modelling efforts.

As a note of interest on pathway topology, it has been suggested that many metabolic and signalling pathways, such as the TLR system, possess a 'bow-tie' or 'hourglass' structure [31,80,91-93]. This is to say that the results from these modelling efforts where pathways are depicted using other notation systems or depicted based only on protein-protein interactions [94], suggest that many signalling pathways possess structures where numerous inputs (ligand/receptor interactions) feed through a small number of 'hub' adaptor molecules to give rise to large and overlapping responses. In this view of signalling pathways proteins common to numerous different signalling systems e.g. MYD88 in the TLR system, are generally viewed as more important than other components in ensuring a network's robustness to perturbation due to their high degree of connectivity. Our model of the TLR system would also support the idea that many of the TLR's all signal through use of a limited number of proteins e.g. MYD88, IRAK4, TIFA, TRAF6, TOLLIP. In each case these proteins act as members of receptor-adaptor complexes propagating the signal induced by ligand binding. In the case of each TLR, the model suggests that further progression of the signal is dependent on a number of MAP3K7 complexes which subsequently go on to activate IKK complex and ultimately NF-κB transcription factors. However, there is also clear evidence linking TLR signalling to the activation of MAPK14 (p38) and MAPK1/3 (ERK) cascades [95,96]. The literature would also suggest that engagement of endosomal TLRs and the MYD88-dependent TLR4 pathway leads to an activation of *IFNB *expression via IRF3 [97,98]. Hence the TLR system acts through numerous signalling networks to bring about the extensive transcriptional changes that are associated with immune activation by LPS or other TLR ligands. The signalling systems used by TLRs are also used by other receptors. For example, the activation of *IFNB *expression via IRF3 is also utilized by ZBP1, DDX58 (RIG-I) and IFIH1 (MDA5) cytosolic DNA/RNA receptors [52,99-101]. Activation of *IFNB *undoubtedly contributes to the transcriptional signature observed when these systems are activated. Further complexity still is evident if we consider that these or indeed other immune responses are all clearly modulated by feedback control [102]. Indeed, a number of negative regulators are observed to be some of the earliest up-regulated genes in the type 1 interferon response measured here e.g. *Socs1/3*, *Prdm1*, *Nfkbiz*. At the same time many feed-forward loops are being established by the up-regulation of transcriptional regulators which go on to activate genes associated with effector systems/pathway modules. Our integrated model of macrophage signalling pathways would therefore suggest extensive cross-talk between pathway modules and transcriptional networks with a high degree of feedback and feed-forward control taking place. It would also predict that many proteins/genes contribute to the networks functional activity and robustness. This is born out by the fact that pathogens are known target many different molecules in their effort to evade or subvert the immune response [103,104], and polymorphisms in numerous proteins has also been shown to be implicated in susceptibility to infectious and inflammatory disease [105-108]. Furthermore, experimental modulation of these systems suggests that their activity is directly regulated by numerous factors acting at different levels [109]. Therefore how real or useful the concept of a bow-tie structure is for such pathways especially with respect understanding their robustness is highly questionable. Indeed the network of molecular interactions that make up the immune system is clearly highly susceptible to perturbation by numerous factors acting at different levels within the network architecture. It has evolved through constant challenges by a diverse range of pathogens. Its robustness relies on the fact that on a population scale its response in different individuals is varied such that what in one may kill, in another may be tolerated.

The task of assembling this diagram has been time consuming and laborious involving 1,000's of hours of work. On the other hand, it summarizes the results of investigations that have taken many times that amount of time to perform and it is difficult to envisage how one could précis this body of work in any other meaningful way. To gain a systems level view of these pathways is to gain an insight into the molecular networks that regulate normal immune function and whose malfunction or manipulation underpins inflammatory and infectious disease. Greater understanding of the overall architecture of the immune system and its susceptibility to deregulation by pathogens and other disease causing agents, should ultimately lead to new strategies and targets for therapeutic intervention. Apart from summarizing decades of research, pathways depicted with formalized graphical notation schemes should aid the communication and comparison of biological data. During a thorough process of internal critiquing sections of the pathway were presented to others who were familiar with the notation scheme but not involved in constructing the pathway presented to them and asked to interpret the biology shown. This process ensured that the interactions of the pathway were not ambiguous in their depiction. Another major incentive for generating pathways with standard notations is to permit the conversion of graphical models into computationally tractable ones, suitable for analysis and simulation. For this purpose we have been exploring the use of signalling Petri nets (SPN) [89] for modelling "flow" in the integrated pathway diagram. The approach is suited to large scale models and the mEPN scheme used to construct the pathway is easily adaptable into a SPN.

For us the exercise of pathway construction has provided a resource for training, pathway modelling, literature/data interpretation, hypothesis generation and as such is now central to our ongoing investigations of macrophage biology. Importantly however, the pathway model presented here also serves as a worked example of how pathways might be represented in a logical, unambiguous and biologist-friendly fashion, whatever the system of interest. What we would like to see and believe is essential, is the support of the wider community in assembling and editing such diagrams. Such efforts are already underway [22-24] and are already providing a vital forum for debate on the known details of pathways in different cell systems. Ideally these efforts will result in detailed models of biological systems that can be shared and assimilated. However, in order to achieve this end pathway models clearly need to be assembled using standard rules and graphical languages. We therefore hope this work will contribute to the ongoing community effort to develop such standards [29].

## Conclusions

The formalised depiction of biological pathways is increasingly recognised as a crucial requirement for the exchange of pathway data, modelling of their activity and systems level interpretation of biological data. However, there are just a handful of worked examples of large pathway diagrams constructed using a formalised graphical modelling language. The model of macrophage signalling and effector pathways presented here is to our knowledge the most comprehensive pathway of its kind published to date. As such it offers a worked example of how large pathway and has also proved to be a testing ground for the mEPN system [31]. When presented in this manner the network reflects the extensive cross-talk between pathway modules and transcriptional networks and high degree of feedback and feed-forward control taking place. This topology does not support the reported bow-tie structure that has been inferred from other pathway modelling efforts.

Although a time consuming and laborious exercise, the act of converting literature derived knowledge into a formalised computational models is essential if we wish to truly gain a systems level understanding of any cellular system. The macrophage model presented here summarizes the results of years of investigations and has allowed the thorough testing of the notation system used to depict it. The hope is that this work will provide a useful resource for others interested in the macrophage and the pathways depicted, and will help contribute to the development of standard graphical depiction in biology.

## Methods

### Data mining, curation and organization

Ongoing analysis of macrophage-related datasets and an interest in consolidating our knowledge of a number of signalling pathways directed our choice of pathways to be mapped (see Figure 1). Public and propriety databases were initially used as resources for data mining, but ultimately all molecular interaction data was sourced from published literature. Manual curation of the literature was performed to firstly evaluate the quality of the evidence supporting an interaction and secondly, to extract the necessary and additional pieces of information required to 'understand' the pathway and construct an interaction diagram. We have drawn pathways based on our desire to model pathways active in a human macrophage and therefore all components have been depicted using standard human gene nomenclature (HGNC). However, our understanding of the pathway components and the interactions between them, have been drawn largely from a consensus view of literature knowledge. As such the pathways presented here are based on data derived from a range of different cellular systems and mammalian species (human and mouse). The following details were captured in an interaction list spreadsheet: PubMed ID (of the paper citing the interaction); the names and official HUGO and Entrez IDs of the interacting components; the nature of the interaction (additional file 2).

### Pathway construction

Individual pathway diagrams focused on a specific area of biology were constructed by teams of 1 or 2 curators who were given a remit to describe a given pathway system using the mEPN scheme [31], generally over a 3-6 month period. Primary curators were junior biologists (MSc students) who were encouraged to use all information resources available to first build up an overall picture of these pathways prior to more detailed analyses and literature-based verification of interactions. Great emphasis was also placed on group work and the need to discuss and justify the information they were attempting to represent to others. Layout was assessed by several curators, as was pathway content and notation usage. Essentially, we attempted to ensure that the graphical depiction of pathway/interactions was intelligible and unambiguous to another individual familiar with the notation scheme. Teams of curators were therefore encouraged to show and discuss their progress with other members of the group on a regular basis.

All pathways have been constructed as directional networks. Interactions between pathway components are drawn using the principles laid down by the mEPN scheme [31] and diagrams assembled according to the workflow described in Figure 1. The current mEPN scheme and a detailed description of the notation scheme and rules for its use are provided in Freeman *et al*., 2010, [31] and http://www.mepn-pathway.org. Individual pathway maps were drawn using the freely available program yEd graph editor (yFiles software, Tubingen, Germany) and later the pathways were integrated using the same software. In order to make these diagrams an information-rich vehicle for conveying details about pathway components and the reactions between them, PubMed IDs supporting the interactions are stored on appropriate edges or nodes within the .graphml version of the diagram, as are URL-links to Entrez gene for each protein or gene component in the pathway and notes from the curators. Due to the nature of pathway construction edges are often moved, deleted then redrawn to optimise the pathway layout and for this reason annotation may also be linked to an appropriate process and/or edge annotation node.

### Pathway Optimization and Integration

Following an initial development period, the focused diagrams went through extensive editing in attempt to unify their notation usage, stylistic qualities and overall appearance. All aesthetics of the pathways (component colours, text font, text size, edge thickness etc.) were standardized between the diagrams. The original pathway diagram [30] was then used as a framework on to which new pathways were 'bolted'. A central rule of the mEPN is that a particular component in a given state may only be represented once in any sub-cellular compartment [28,30,31] Thus when integrating the diagrams a crucial step was to identify, using the interaction and component lists, overlap between pathway members in the individual diagrams. Connections could then be built between the individual pathways based on shared pathway members and common interactions. For example, a number of the systems of interest feed into the NF-кB system including the TLR and non-TLR pathogen detection receptor signalling, TNF-receptor activation, apoptosis and MAPKinase signalling. If the representation of interactions differed between individual diagrams they were re-examined by going back to the literature. Furthermore, annotations and curators notes were moved and preliminary layouts optimized. Depicting this interconnectivity ultimately leads to numerous challenges in arranging the layout of the diagram. This was particularly acute when laying out the integrated diagram. A significant leap forward was made with the realization that however 'optimized' the layout of the diagram it was too large to be displayed in a readable format on a single page (as had always been the aim when working on a smaller scale when trying to produce a 'publication ready' layout). With this in mind we could be more free with our use of space and in the final layout, pathway 'modules' consisting of numbers of connected nodes involved in a similar system are separated out. This has the effect that more space is available to run tracks of parallel edges between modules and subsequent additions or editing are easier to perform. Following the integration of TLR system with the original diagram, the NF-κB, non-TLR and proteosome maps were added sequentially according to the same principles. The fully integrated map then underwent an extensive series of layout optimizations in order to bring visual clarity to final product.

### Pathway Overlay of the Transcriptional Response Analysis of Mouse Bone Marrow Derived Macrophages to Interferon beta Treatment

Analysis of a timecourse of interferon-beta (Ifn-β) treatment of mouse bone marrow derived macrophages (BMDMs) was used as an example of data integration onto the macrophage signalling pathway using BioLayout *Express*^3D. ^Mouse BMDM preparation, cell culture, and cytokine (Ifn-β) treatment was performed as described in the data sets GEO submission; *accession no GSE20403*. Expression data (available at GEO, accession GSE20403) was normalized using the RMA package within the Affymetrix Expression Console software and annotated. Transcripts which might be considered to be differentially expressed at each time point compared to 0 h were identified using the Empirical Bayes function within the Bioconductor [110] package of the *R *statistical program using a 1.5 fold cut-off and a p-value of 0.05. According to this analysis 2,300 transcripts were considered to be differentially expressed. Data corresponding to these transcripts was then loaded into the network visualization tool BioLayout *Express*^3D ^[76,77] using a Pearson correlation cut-off of 0.9 to filter edges. The resultant network graph of 2,045 nodes (connected by 92,947 edges) was clustered using the graph-based clustering algorithm MCL [111] set at an inflation value of 2.2 resulting in 33 clusters (with greater than 5 nodes). Manual inspection of the clusters revealed 21 clusters of interest i.e. their expression profile was consistent with the genes being regulated by Ifn-β. Resulting clusters represent patterns of co-expression amongst transcripts in the network graph. The clustered data was then exported as "class-sets" and overlaid onto the macrophage pathway diagram within BioLayout *Express*^3D ^using the "import-class-sets" functionality of the tool. Where genes are present in clusters of co-expressed transcripts and are also present in the pathway they can then be visualized and highlighted on the pathway.

## Authors' contributions

SR was instrumental in the development of the mEPN scheme, oversaw the assembly of TLR signalling and integrated pathway diagrams, contributed to the development of the notation system, performed the Ifn-β timecourse study, oversaw the labelling of the RNA for microarray processing, and was a primary author of the manuscript; NM played a major role in the integration of the final pathway diagram and together with SM, helped redefine the TLR signalling pathway; PAL constructed the initial interferon pathways and oversaw the construction of the non-TLR pathogen detection by GE and MO; KR and SW contributed to development of the pathway notation and standardization of pathway data collection; YC and MC assembled the NF-κB pathway; AT (A Turnbull) assembled the antigen presentation, ubiquitin and proteasome pathways; AT (A Theocharidis) has been developing the program BioLayout *Express*^3D ^to enhance its capabilities to support these pathways for visualization and data integration; PG started manual curation of pathways and the original development of the EPN scheme and supported the current development; finally TCF oversaw and contributed to the pathway construction, orchestrated the development of the mEPN scheme, has directed the development of improved computational resources for these pathways and drafted the manuscript. All authors read and approved the final manuscript.

## Supplementary Material

Additional file 1 **Integrated Map of Macrophage Pathways_graphml-Version**. This file can be opened, viewed and edited by users using the freely available graph-editor software yEd (yFiles software, Tubingen, Germany). yEd can be downloaded at http://www.yworks.com/en/downloads.html#yEd where full downloading instructions are described. PubMed IDs supporting the interactions of the pathway are stored on appropriate edges and nodes within this .graphml file. Once an edge is selected the PubMed ID may be viewed within the descriptions tab of the properties box for that edge or node. Individual proteins have a url link to their Entrez gene entry. Furthermore some (complicated) processes also have additional notes to describe the events and can be found under the description tab of properties and when hovering over the node. Click here for file

Additional file 2 **List of Pathway Interactions and Supporting Publications**. Interactions included in the pathway map are listed. Official HGNC (human gene nomenclature committee) gene symbols are used to name the interacting components and Entrez gene IDs are also provided. Interacting components are named as individual genes/proteins and also as they appear on the pathway where they may be interacting as part of a protein complex or with a modification (e.g. Phosphorylation). Interactions are described as they appear on the pathway, sub-cellular location and the NCBI-PubMed ID for supporting literature is provided. There are also columns to incorporate additional information (cell type, analytic method etc) where possible. Click here for file

Additional file 3 **Integrated Map of Macrophage Pathways_pdf-Version**. A high resolution .pdf file for viewing the integrated map of macrophage pathways. Click here for file

Additional file 4 **Integrated Map of Macrophage Pathways_html-Version**. A high resolution flash .html version of the integrated macrophage pathway for viewing in a web browser. Click here for file

Additional file 5 **Integrated Map of Macrophage Pathways in 3D-or-2D View**. The integrated macrophage pathway can be viewed in the network visualisation tool BioLayout *Express*^3D^, in both 2 and 3-dimentions and also using the original graph co-ordinates as the .graphml file. By choosing the appropriate class-type (found within class tab of graph properties box) the nodes can be coloured according to assigned classes. By default nodes are coloured and shaped according to "node-type" (e.g. proteins, genes, complexes, process nodes) using a 3D-notation scheme described at http://www.mepn-pathway.org. Nodes may also be coloured according to sub-cellular location by assigning the "Cellular location" class. It is also possible to import other class-sets of interest; for example information about a components regulation/behaviour in a dataset. Within BioLayout *Express*^3D ^one can easily follow the flow of information through the pathway by selecting parent or children nodes of a component of interest to highlight them. BioLayout *Express*^3D ^software is freely available and can be downloaded at http://www.biolayout.org/ Click here for file

Additional file 6 **Overlay of Ifn-β Regulated Targets onto the Integrated Map of Macrophage Pathways**. Components within clusters of co-ordinately expressed transcripts following Ifn-β stimulation were exported as class sets from a mouse expression graph generated in BioLayout *Express*^3D ^and imported onto the 'human' macrophage pathway diagram (.layout file) within BioLayout *Express*^3D^. Pathway components that are regulated by Ifn-β are enlarged and coloured according to their behaviour within the dataset and all other nodes are coloured grey, in this way allowing the easy identification and visualisation of regulated components. BioLayout *Express*^3D ^software is freely available and can be downloaded at http://www.biolayout.org/ Click here for file
