# Supplementary material for: Construction of a large scale integrated map of macrophage pathogen recognition and effector systems
Source: BMC Syst Biol. 2010 May 14;4:63. doi: 10.1186/1752-0509-4-63 (PMC2892459; doi:10.1186/1752-0509-4-63)
Supplement: Additional file 3 — Integrated Map of Macrophage Pathways_pdf-Version. A high resolution .pdf file for viewing the integrated map of macrophage pathways. [file 1752-0509-4-63-S3.PDF]

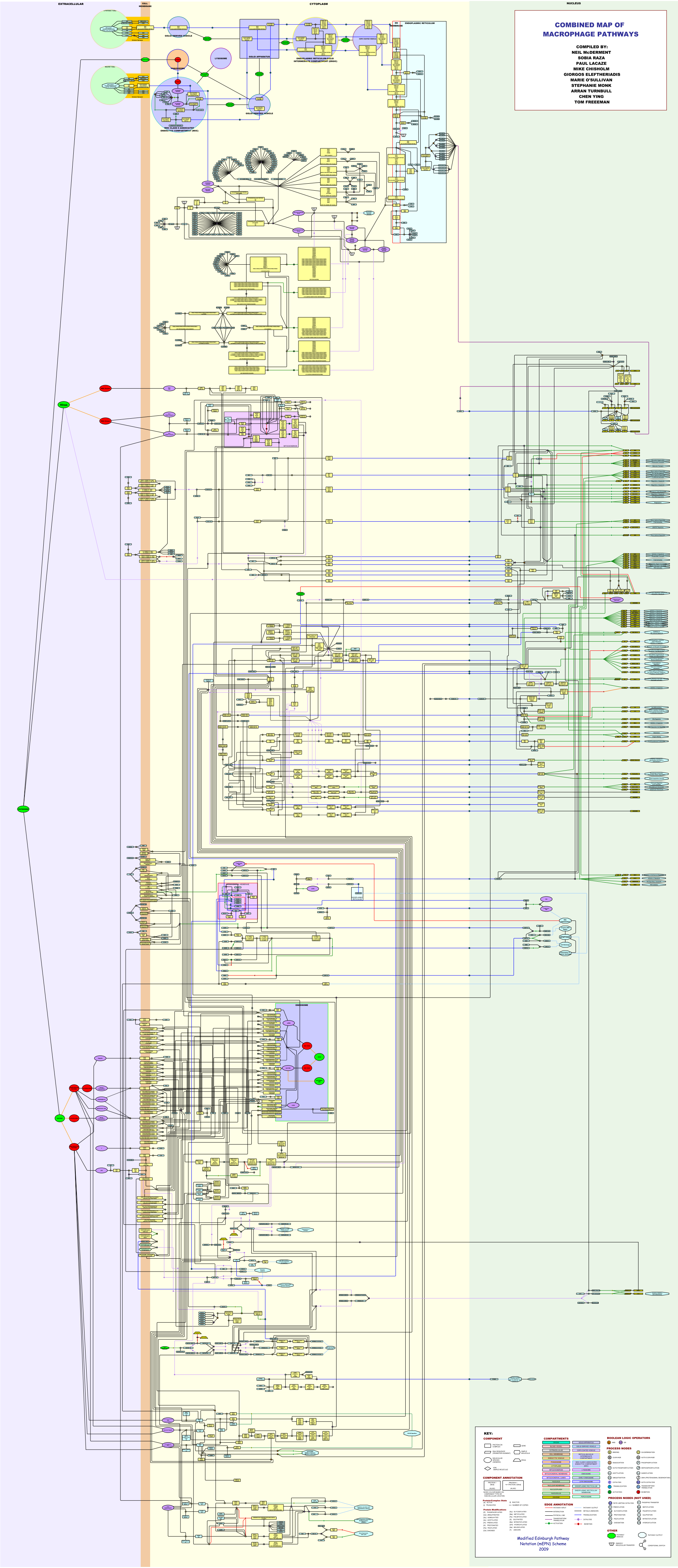

EXTRACELLULAR
CELL MEMBRANE
CYTOPLASM
NUCLEUS
COMBINED MAP OF
MACROPHAGE PATHWAYS
COMPILED BY:
NEIL McDERMENT
SOBIA RAZA
PAUL LACAZE
MIKE CHISHOLM
GIORGOS ELEFTHERIADIS
MARIE O'SULLIVAN
STEPHANIE MONK
ARRAN TURNBULL
CHEN YING
TOM FREEEMAN
KEY:
COMPONENT ANNOTATION
EDGE ANNOTATION
PROCESS NODES (NOT USED)
OTHER
Modified Edinburgh Pathway
Notation (mEPN) Scheme
2009
